# Integrating Habitat Suitability and Quality Assessments to Identify Conservation Priorities for *Cycas panzhihuaensis*

**DOI:** 10.3390/plants15040670

**Published:** 2026-02-23

**Authors:** Yuanfeng Yang, Yuting Ding, Xuefeng Peng, Juan Wang, Peilong Li, Mengjie Wu, Ying Zhang, Xing Liu, Peihao Peng

**Affiliations:** 1 College of Geography and Planning, Chengdu University of Technology, Chengdu 610059, China; yuanfengyang125@gmail.com (Y.Y.); 2023010044@stu.cdut.edu.cn (Y.D.); idwangjuan@cdut.edu.cn (J.W.); 18236340110@163.com (P.L.); mengjiewu0604@163.com (M.W.); 529213949@139.com (Y.Z.); llxxxlxx@163.com (X.L.); 2 Institute of Mountain Hazards and Environment, Chinese Academy of Sciences, Chengdu 610213, China; piceapen@163.com; 3 University of Chinese Academy of Sciences, Beijing 100049, China

**Keywords:** *Cycas panzhihuaensis*, MaxEnt, InVEST, suitable habitat, habitat quality, hotspots

## Abstract

This study assessed the conservation priorities for *Cycas panzhihuaensis*, a relict plant endemic to the dry-hot valleys of the Jinsha River, by integrating habitat suitability prediction with habitat quality evaluation. We used the MaxEnt model to identify its potential distribution and key environmental drivers and the InVEST model to evaluate habitat quality and degradation risk within the study area. Conservation priorities—categorized as hotspots, transition zones, and coldspots—were delineated by overlaying suitability classes with habitat quality levels. Spatial clustering of hotspots was examined using global spatial autocorrelation analysis. The results indicate that: (1) The highly suitable habitat for *C. panzhihuaensis* covers an area of 799.12 km^2^, primarily concentrated in the dry-hot valleys of the Jinsha, Yalong, and Anning Rivers. January land surface temperature was the most significant environmental determinant of its distribution (contribution: 36.1%). (2) The overall habitat quality of the study region was relatively low (mean: 0.38), with a moderate risk of degradation. Areas of severe degradation spanned 14,629.31 km^2^ (26.10% of the total area), largely coinciding with the river valleys and showing substantial overlap with the species’ suitable habitat. (3) The identified conservation hotspots (799.63 km^2^) exhibited a moderate and statistically significant positive spatial autocorrelation (global Moran’s *I* = 0.326). This integrated approach provides a spatially explicit framework for conservation planning, offering valuable insights applicable to other rare species in human-impacted landscapes.

## 1. Introduction

Species habitat distribution and quality assessment form a critical scientific foundation for biodiversity conservation and ecological spatial planning [1,2]. Under the dual pressures of global climate change and human activities, many species are facing severe challenges of habitat loss, degradation, and fragmentation [3]. In this context, accurately identifying suitable habitats for species and evaluating their threat status have become essential prerequisites for developing effective conservation strategies. The dry-hot valley region in southwestern China, characterized by its unique yet fragile ecosystems, represents both hotspots and critical areas for biodiversity conservation [4].

*Cycas panzhihuaensis* L. Zhou & S. Y. Yang is a relict plant species with significant evolutionary importance within the family Cycadaceae, which has persisted for over 200 million years [5]. Not only is it a xerophytic rock-dwelling species endemic to the Jinsha River dry-hot valleys, but it also serves as a dominant woody plant in the region’s shrub communities [6]. *C. panzhihuaensis* predominantly inhabits steep slopes composed of calcareous rock, shale, and sandstone (Figure S1a–d) [7]. It plays a vital ecological role in slope stabilization, soil and water conservation, and the maintenance of microhabitat diversity [8]. Despite its significant ecological value, this species continues to face severe survival threats. Its slow and protracted reproductive cycle, combined with harsh habitat conditions, severely limits natural regeneration. Historically, over exploitation has led to a sharp decline in population size, while ongoing anthropogenic disturbances exacerbate habitat fragmentation, further deteriorating its conservation status [9]. In 2022, the species was classified as Vulnerable on the IUCN Red List [10].

Current research on *C. panzhihuaensis* has predominantly focused on its physiological responses, population genetics, and phylogeography. For instance, Xia Zhining et al. [11] investigated the physiological and biochemical responses of *Cycas bifida* (Dyer) K. D. Hill and *C. panzhihuaensis* to freezing stress, revealing that the semi-lethal low temperature for *C. panzhihuaensis* is −2.05 °C. Yu Jiao et al. [12] elucidated its lipid metabolic response mechanisms under combined stress conditions, demonstrating a relatively strong tolerance to individual drought stress but heightened sensitivity to combined drought and high-temperature stress. In terms of edaphic factors, Long Cheng et al. [13] found that soil available potassium and pH exert the greatest influence on the population distribution of *C. panzhihuaensis*, noting that the roles of soil available nitrogen and available phosphorus are expected to increase relative to available potassium as forest succession progresses. Studies on propagation, such as those by Yang Yongqiong et al. [14] and Wu Qunyu et al. [15] on artificial and asexual reproduction, have provided crucial insights for population genetics and conservation. Furthermore, Hao Yunqing et al. [16] conducted a floristic study of associated seed plants, revealing communities predominantly composed of tropical elements with abundant pantropical components, which, along with pronounced dry-hot valley characteristics, indicates strong tropical affinities and ancient origins. In contrast, research integrating its habitat suitability, quality assessment, and the relative influence of environmental versus anthropogenic factors remains scarce.

Species Distribution Models (SDMs) and Ecosystem Service Assessment Models represent two pivotal tools for elucidating the relationships between species and their environments and for evaluating habitat quality. Among the mainstream SDMs, the Maximum Entropy (MaxEnt) model effectively predicts potential suitable habitats for species based on occurrence records and environmental variables. Renowned for its robustness with small sample sizes, rapid computational performance, and high operational efficiency. MaxEnt has been widely applied in studies predicting species’ potential suitable habitats, such as those on *Ageratina adenophora* (Spreng.) R. M. King&H. Rob [17], *Psammosilene tunicoides* W. C. Wu & C. Y. Wu [18], *Vaccinium duclouxii* (H. Lév.) Hand.-Mazz. and *Vaccinium fragile* Franch. [19], as well as *Quercus cocciferoides* Hand.-Mazz. [20], among others.

The InVEST model is designed to simulate changes in the physical quantity and value of ecosystem services under different land use scenarios, thereby providing a scientific basis for balancing the benefits and impacts of human activities [21]. The habitat quality module within InVEST can quantitatively assess the extent of habitat degradation and overall habitat quality by evaluating the impacts of anthropogenic threats such as land-use change. In recent years, numerous scholars in China and abroad have employed the InVEST model to conduct in-depth assessments of habitat quality, further exploring the effects of land-use patterns on habitat quality from a spatial configuration perspective [22,23,24].

However, existing studies predominantly apply these models in isolation, failing to effectively integrate predictions of species’ suitable habitats with their actual threat status [23,24,25]. This limitation makes it difficult to systematically address the following core scientific questions: How can we accurately identify key habitats that possess both ecological suitability and high habitat quality? How can we quantify the coupled effects of multiple environmental factors and anthropogenic threats on the survival space of rare species? This may lead to the misidentification of conservation priorities, thereby compromising the scientific basis of decision-making.

To address this limitation, we integrate the results of the MaxEnt and InVEST models, aiming to clearly answer the following questions through a multi-model integration approach: (1) How do the potentially suitable distribution areas of *C. panzhihuaensis* spatially align with actual high-quality habitats? (2) What are the relative contributions of different anthropogenic threat sources to its habitat degradation? (3) How can we establish a comprehensive conservation priority system that balances suitability, quality, and vulnerability? This approach not only identifies environmentally suitable areas but also directly assesses their habitat quality and threat levels [26,27], thereby allowing for a more precise delineation of “high-suitability, high-quality” hotspots.

Within this framework, although recent research has conducted pioneering analyses of habitat suitability and conservation prioritization for *C. panzhihuaensis* using MaxEnt, Geodetector, and Zonation [28], their prioritization approach primarily relies on optimizing distribution probabilities and fails to directly quantify the degradation risks posed by human disturbances to habitats. To address the questions raised above, this study builds upon their species distribution data and integrates the MaxEnt model with the habitat quality module of the InVEST model to construct a comprehensive framework that simultaneously assesses potential suitability and actual habitat quality. We have further refined the environmental variable system by incorporating more comprehensive soil physicochemical properties and higher-resolution climatic factors. Additionally, we emphasize the use of the InVEST model to simulate habitat degradation under multiple threat sources, thereby identifying key areas that exhibit both high suitability and relatively high habitat quality.

This framework transcends the limitations of single-distribution predictions by enabling the direct assessment of current habitat quality and threats, systematically addressing the core questions posed by this research, and providing a more precise foundation for formulating targeted conservation measures aimed at mitigating degradation and enhancing habitat quality.

In summary, as a species shaped by the combined effects of climate, topography, soil, and human activities, *C. panzhihuaensis* offers an ideal model for investigating the intrinsic mechanisms through which these variables drive species distribution patterns.

## 2. Results

### 2.1. Assessment of Maxent Model Accuracy

The MaxEnt model results showed an average AUC value of 0.964 for *C. panzhihuaensis* across the study area, indicating that the model achieved an excellent level of predictive accuracy [29]. The AUC plot is provided in Figure S2.

### 2.2. Main Factors Affecting the Distribution of C. panzhihuaensis

**Figure 1:**
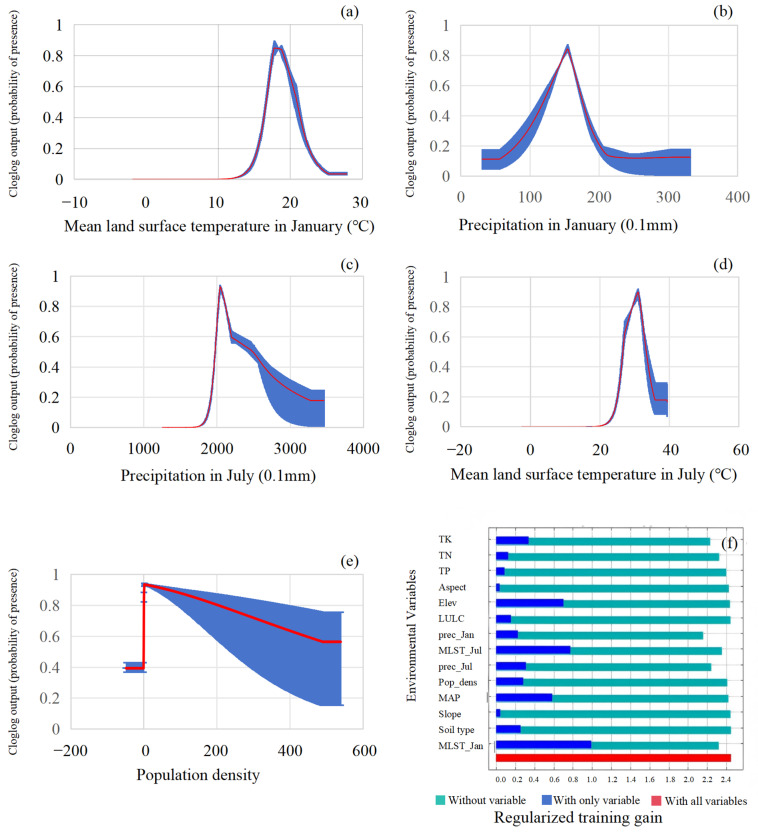
Response curves of the five main influencing factors and the results of the slicing method for the suitable habitat of *C. panzhihuaensis.* (The red curve represents the mean occurrence probability across all MaxEnt model runs, and the blue area (±SD) indicates the range of response variation). (**a**) Mean land surface temperature in January; (**b**) Precipitation in January; (**c**) Precipitation in July; (**d**) Mean land surface temperature in July; (**e**) Population density; (**f**) Jackknife analysis of the relative influence of major environmental variables on the distribution of *C. panzhihuaensis*.

A jackknife analysis was performed to assess the importance of individual environmental variables (Figure 1f). The results revealed that January land surface temperature, July land surface temperature, and elevation were the key drivers of the potential distribution for *C. panzhihuaensis*. Furthermore, the variation in habitat suitability was analyzed based on single-variable response curves (control-variable approach) to identify the critical ranges of environmental variables corresponding to high-suitability areas [30] (suitability > 0.57) (Figure 1a–e). The top five environmental factors contributing to the model were in descending order, January Land Surface Temperature (36.1%), January Precipitation (12%), July Precipitation (10%), July Land Surface Temperature (9.6%), and Population Density (7.8%) (Table 1). The response curves indicated that the probability of *C. panzhihuaensis* occurrence peaked under the following approximate conditions: January land surface temperature of ~19 °C, January precipitation of ~15.5 mm, July precipitation of ~210 mm, a July land surface temperature of ~32 °C, and a population density approaching zero (per 900 m^2^). These results suggest that *C. panzhihuaensis* is sensitive to low temperatures, preferring a warm winter [31], moderate precipitation, and minimal human disturbance within the dry-hot valley habitat [28].

### 2.3. Potential Suitable Habitat of C. panzhihuaensis Under Contemporary Climate

**Figure 2:**
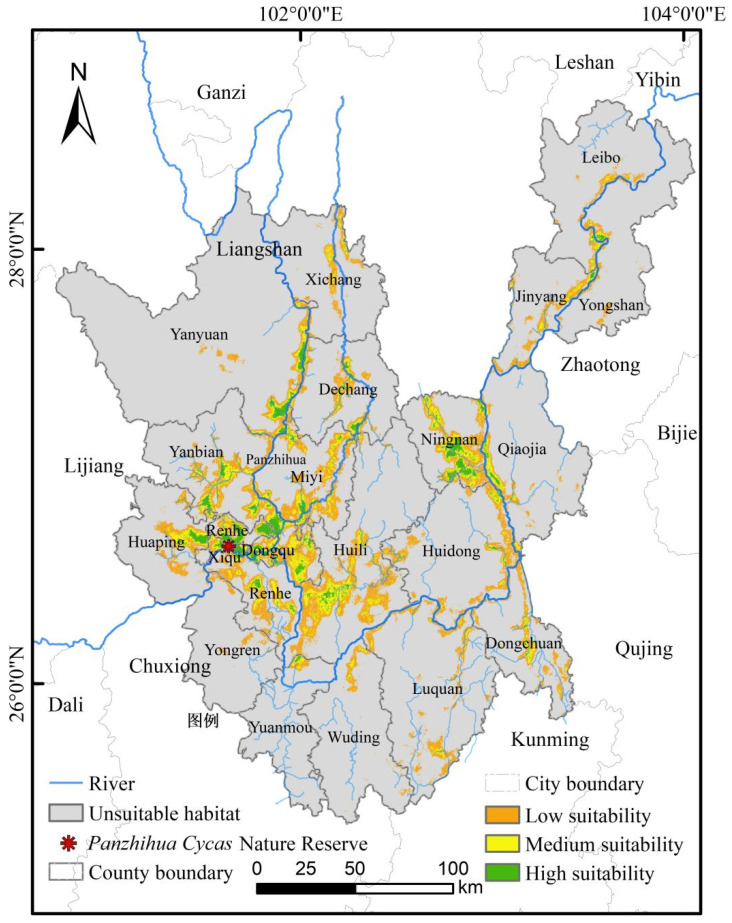
Schematic distribution map of suitable habitat for *C. panzhihuaensis* under 2020 climatic conditions predicted using the MaxEnt model.

The suitable habitat for *C. panzhihuaensis*, classified using the Natural Breaks method, is shown in Figure 2. Suitable areas are primarily concentrated in the valleys of the Jinsha River, Yalong River, and Anning River. The habitat suitability was categorized into four levels: non-suitable [0, 0.07], low-suitable [0.07, 0.26], medium-suitable [0.26, 0.57], and high-suitable [0.57, 1.00] [18]. The total area of highly suitable habitat is 799.12 km^2^, medium-suitable habitat covers 1986 km^2^, low-suitable habitat spans 5322.26 km^2^, and non-suitable areas comprise 47,957.21 km^2^. The largest contiguous patch of highly suitable habitat is located at the junction of Dong District, Xi District, and Renhe District in Panzhihua City, which coincides with the current location of the *C. panzhihuaensis* National Nature Reserve.

### 2.4. InVEST Model Habitat Quality Index

**Figure 3:**
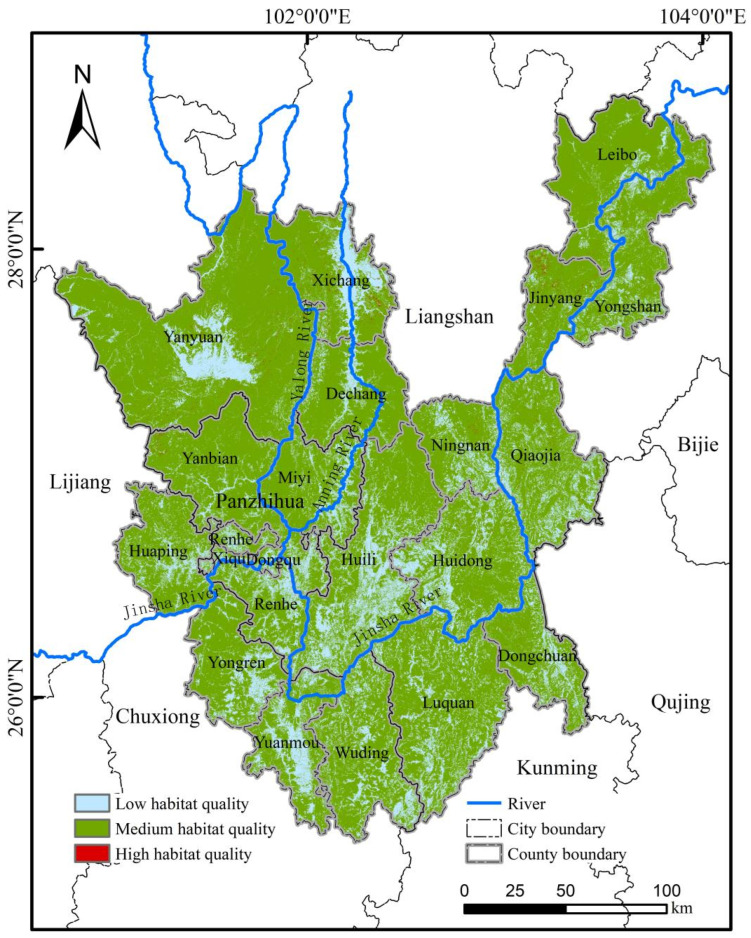
Habitat quality distribution of the study area in 2020 derived from the InVEST model.

The InVEST model results indicated an average habitat quality index (HQI) of 0.38 for the study area, suggesting an overall low level of habitat quality. Using the Natural Breaks method, the HQI was classified into three levels: [0, 0.24], [0.24, 0.69], and [0.69, 1.00], representing low, medium, and high habitat quality for *C. panzhihuaensis*, respectively (Table 2). High quality habitats were present in all counties/districts within the study area but were severely fragmented and of limited extent, covering only 0.25% of the total area (141.45 km^2^). This extreme fragmentation makes it difficult to plan for contiguous protected areas (Figure 3). In contrast, medium-quality habitats comprised a vast, contiguous area and are thus recommended as the priority for conservation and reintroduction efforts.

### 2.5. Habitat Degradation Risk Index

**Figure 4:**
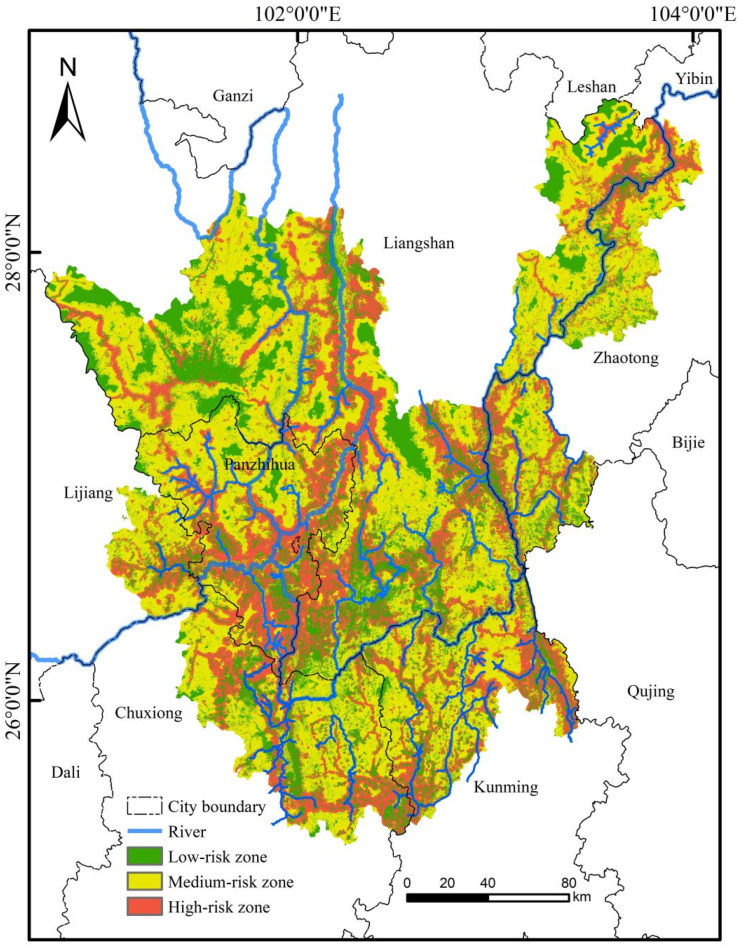
Habitat degradation risk distribution of the study area in 2020 derived from the InVEST model.

The habitat degradation risk index across the study area spanned from 0 to 0.66, with an average of 0.17, reflecting a moderate degradation risk. This index was further categorized into low, medium, and high degradation risk levels via the Natural Breaks classification (Figure 4). Subsequently, the spatial extent and proportional coverage of each risk category were quantified (Table 3). Highly degraded areas covered 14,629.31 km^2^, accounting for 26.10% of the total study area. They were primarily located in river valleys, which are traditional hubs of intensive human activity, and exhibited a pronounced spatial overlap with the suitable habitat range of *C. panzhihuaensis*.

### 2.6. Spatial Autocorrelation Analysis of Habitat Conservation Hotspots

The spatial autocorrelation analysis of the habitat hotspots, derived from the intersection of the MaxEnt model (suitable habitat) and InVEST model (high habitat quality) results, yielded a global Moran’s *I* index of 0.326. This indicates a moderate and statistically significant positive spatial autocorrelation for *C. panzhihuaensis* habitat hotspots, suggesting a well-clustered distribution pattern (Table 4).

The total area of these identified hotspots is 799.63 km^2^, accounting for 1.43% of the total study area. The primary land use types within these hotspots include grassland, forest, cropland, and bare land.

In terms of absolute area, the largest extents of hotspots are found in Ningnan County (126.22 km^2^), Yanbian County (128.93 km^2^), and Renhe District (132.06 km^2^). However, relative to the size of the administrative unit itself, Xiqu District of Panzhihua City contains the highest proportion of hotspots, covering 42.45% of its total area. For comparison, the total area of habitat coldspots is 5319.68 km^2^ (9.49% of the study area), and that of transitional zones is 1988.08 km^2^ (3.55%).

**Figure 5:**
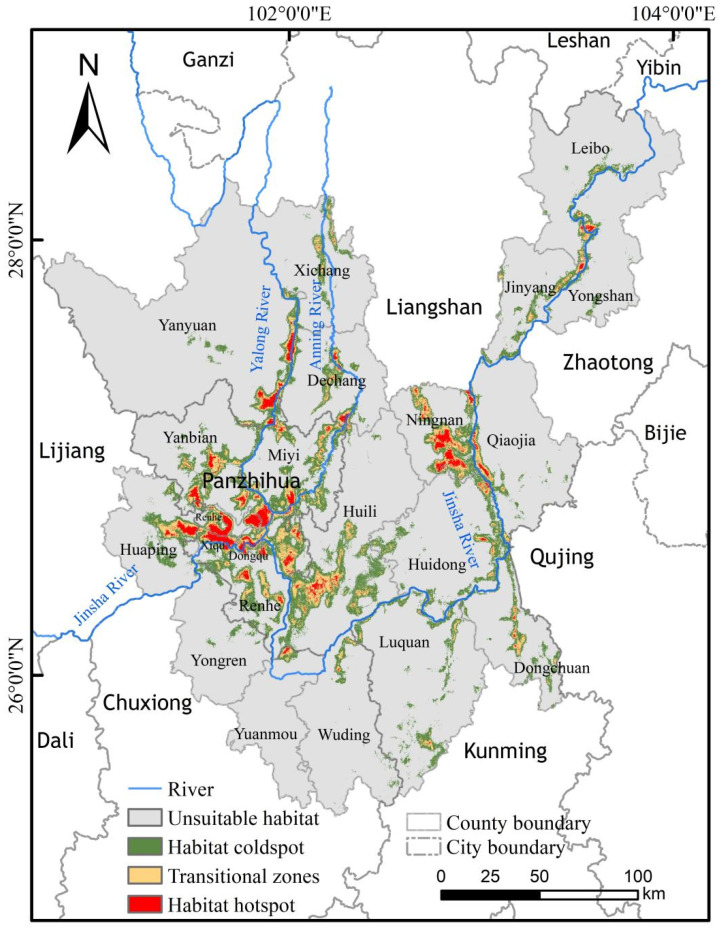
Habitat conservation coldspots, transitional zones, and hotspots for *C. panzhihuaensis* in the study area.

Within the designated hotspots, a distinct spatial disjunction was observed between the high-suitability zones for *C. panzhihuaensis* and the areas of high habitat quality. The spatial overlap between these two high-value categories was minimal, covering an area of only 100 m^2^. In contrast, the predominant pattern identified was “High suitability—Medium habitat quality,” primarily distributed along river corridors. The associated ecosystem types included dry grasslands, valley shrublands, and lower-montane forests. A clear spatial gradient was evident, transitioning sequentially from coldspots to transitional zones and finally to hotspots, with the hotspots located closest to the rivers (Figure 5). The priority conservation hotspots identified in this study are recommended as potential sites for the reintroduction of *C. panzhihuaensis* populations.

## 3. Discussion

This study employed the MaxEnt ecological niche model to delineate suitable habitats for *C. panzhihuaensis* and utilized the habitat quality module of the InVEST model to assess habitat quality. The suitability and habitat quality results were classified, intersected, and subsequently categorized into habitat conservation hotspots, transitional zones, and coldspots. By integratively considering the spatial distribution of *C. panzhihuaensis* under the combined effects of natural conditions and anthropogenic disturbances, this approach more accurately identifies priority conservation areas suitable for the species’ survival. The findings provide a crucial scientific basis for formulating science-based and efficient conservation strategies.

### 3.1. Factors of Distribution Patterns

The results of this study indicated that January Land Surface Temperature was the primary determinant of suitable habitat distribution (contribution rate: 36.1%), suggesting that low temperature represents the most critical limiting factor for the survival and distribution of *C. panzhihuaensis* [11,32]. The relatively high land surface temperature in January within the region ensures that *C. panzhihuaensis* avoids frost damage during winter. Furthermore, the extended period of relatively high temperatures throughout the year contributes to a prolonged growth and development cycle for this species. The habitats of *C. panzhihuaensis* are predominantly concentrated in the dry-hot valleys of major river systems, including the Jinsha River, Yalong River, and Anning River [9]. Dry-hot valleys are deeply incised valleys predominantly distributed within the Hengduan Mountain region of southwestern China. Their defining characteristics include abundant thermal energy, scarce moisture, distinct seasonal alternation between dry and wet periods, consistently high annual temperatures, and intense solar radiation [33], which stand in stark contrast to the humid climate of the surrounding areas. Collectively, these three major rivers form a distinctive “thermal corridor,” which has created an ideal refugium for *C. panzhihuaensis* [20]. Corridor enabled the species to survive here as a glacial refugium following the Quaternary ice ages and persist to the present day. Furthermore, the inclusion of population density as a key variable underscores the profound impact of anthropogenic activities on its contemporary distribution pattern. During the Three-Year Natural Disaster period, local farmers in areas such as Xiqu District of Panzhihua City and Huili Mugu harvested the stems and leaves of *C. panzhihuaensis* for human consumption or as fodder for pigs, causing localized damage to the population. However, the level of damage attributable to subsistence harvesting was relatively limited. Following the economic reforms and opening-up policies initiated in the late 1970s, accelerated urban expansion coincided with the growing recognition of the ornamental value of cycads. This led to widespread illegal harvesting and poaching between the 1970s and 1990s, resulting in a precipitous decline in population numbers [9]. Concurrently, urbanization within the region and the expansion of contemporary agricultural economies (e.g., mango and Sichuan pepper cultivation) in the valley areas have collectively exacerbated the loss and fragmentation of its suitable habitat [34].

### 3.2. Spatial Decoupling of Habitat Suitability and Quality

Analysis of the hotspots revealed a significant spatial mismatch between high-suitability zones for *C. panzhihuaensis* and areas of high habitat quality. The high-suitability zones identified by the MaxEnt model are predominantly concentrated in valley areas with intense human activity. In contrast, the high-quality habitats assessed by the InVEST model, which cover a total area of only 141.45 km^2^, are severely fragmented. The spatial overlap between these two categories is minimal, amounting to merely 100 m^2^. Habitat quality shows a direct negative correlation with human disturbance: regions characterized by higher population density, more frequent anthropogenic activities, and proximity to threat sources exhibit significantly lower habitat quality compared to other areas [24,35,36]. The optimal habitats for *C. panzhihuaensis*—open woodland grasslands, valley shrublands, and oak forests in the lower forest stratum [9]—coincide precisely with the areas most favored for human cultivation, road construction, and settlement in this region. The expansion of construction land, road networks, and cropland has directly encroached upon these pristine habitats. Consequently, even in areas where environmental conditions remain suitable, the habitat quality suffers severe degradation due to anthropogenic pressures [37]. Remote sensing imagery reveals that the areas surrounding most historical occurrence points have been converted to cropland and economic forest plantations (e.g., mango, Sichuan pepper, pomegranate). This transformation indicates that the microhabitats at these sites have been degraded and altered by human activities.

### 3.3. Hierarchical Zoning Conservation Strategies

Hotspots (characterized by high suitability and medium-to-high habitat quality) should be designated as core zones for prioritized conservation and stringent management [38]. Priority should be given to contiguous high-suitability areas along the main stem of the Jinsha River (e.g., the junction of the three districts in Panzhihua City, eastern Huaping County, southern Ningnan County, Kahaluo Township in Leibo County, and southwestern Huili City) and within the Yalong River valley (e.g., the border area between Dechang County and Yanyuan County). We recommend that the existing *C. panzhihuaensis* National Nature Reserve serve as the core, with the incorporation of adjacent hotspots into the statutory protected area system to expand its conservation coverage [39]. Building on this foundation, a systematic reintroduction and population augmentation plan should be developed. This plan should fully utilize the in situ populations within the protected area as a high-quality germplasm resource bank. Introducing individuals from this source to hotspots outside the reserve is expected to enhance seedling survival rates [40]. In the hotspots with favorable ecological conditions in eastern Huaping County, the establishment of a new nature reserve or conservation area should be planned. This would serve to protect the local native Cycas population, forming a collaborative and interactive conservation network with the existing reserve. Such a network would enhance gene flow among populations [41]. To safeguard core habitats, it is imperative to impose strict limitations on new development activities, particularly mining, road construction, and agricultural expansion, within and around the protected area. Furthermore, ground patrol efforts must be significantly amplified in both intensity and technological sophistication to ensure habitat security and stability [42].

For hotspots located outside formal protected zones, which pose a distinct management challenge due to their decentralized nature, this study proposes a novel framework integrating relocation protection and community collaborative conservation [43]. This approach is designed to leverage existing local resources, aiming to simultaneously facilitate population restoration and incentivize community participation in sustainable development [44,45]. In these areas, rampant historical poaching has led to the local extinction of wild populations, although the habitats themselves remain suitable. Field surveys have revealed that a substantial number of living *C. panzhihuaensis* individuals are still preserved within local communities. Furthermore, communities have mastered techniques for their successful cultivation and for facilitating natural population regeneration. A viable strategy involves relocating these currently scattered individuals (e.g., from household gardens) back to their original native habitat and selecting new sites for planting that are accessible for human management. Due to their proximity to villages, these sites facilitate daily monitoring and care by local residents, thereby significantly enhancing transplant survival and long-term preservation rates [46]. A detailed electronic database should be established to catalog both the currently extant cultivated individuals and those to be relocated and planted for conservation purposes in the future [47]. Technical guidance should be provided by specialized institutions, while the day-to-day management and custodianship should be entrusted to local community farmers [48]. This initiative aims to establish a community-based decentralized conservation network to foster a favorable environment for the recovery of *C. panzhihuaensis* [49].

Transitional zones function as ecological corridors that connect hotspots, facilitating species dispersal, while also providing buffer space for core populations. Typically, these zones are distributed around the periphery of hotspots, encircling them. Consequently, many hotspots become directly adjacent to threat sources—such as cropland and roads—within or adjacent to these transitional zones, exposing them to immediate disturbance risks [36]. The primary focus for the conservation management of transitional zones should be on the planning and restoration of ecological corridors. Specifically, those areas adjacent to core zones should be designated as buffer strips [50]. Within this zone, land-use practices compatible with the conservation of *C. panzhihuaensis* should be promoted. For example, communities should be guided to develop agroforestry or non-timber forest product economies as alternatives to deforestation for cultivation, thereby enhancing the ecological security of hotspots [51]. Concurrently, assisted natural regeneration strategies and targeted management measures (e.g., reducing grazing pressure, controlling invasive species) should be implemented to promote the recovery of native vegetation. This will help to reconnect fragmented habitats, thereby creating conduits for pollen flow, seed dispersal, and individual movement [52].

Coldspots constitute a large, peripherally fragmented zone located beyond the transitional zones. In this study, the delineated coldspots are designated as zones for guided development, aiming to foster an intensive, clustered development pattern. This strategy is intended to prevent new encroachment and fragmentation of transitional zones and hotspots. The goal is to achieve the objective of “Sustainable Development” without compromising regional economic growth [53]. The focus of conservation and restoration efforts should be precisely on the dry valley ecosystem itself, rather than on simplistic afforestation [54]. Priority should be given to the use of native shrub and grass species that are associated with *C. panzhihuaensis*—such as *Saccharum fallax* and *Quercus cocciferoides*, which are characteristic of the sun-exposed dry valley shrubland ecosystem—in order to reconstruct the essential microhabitats upon which it depends [55].

The spatial pattern of conservation priority areas identified in this study confirms the predictions of Ding et al. [28], demonstrating that regions of high conservation value are predominantly distributed in a belt-like pattern along the dry-hot valleys of the Jinsha and Yalong Rivers. This reaffirms the critical role of this thermal corridor for the survival of *C. panzhihuaensis*. Moreover, this spatial pattern shows substantial overlap with the core areas of the Evolutionarily Significant Units (ESUs) delineated by Xiao et al. [56], particularly within the major PZH, SL, and PDH units (Figure S3). The habitat hotspots mapped in this study thus provide a spatially explicit basis for selecting core protection sites.

Furthermore, our results offer an ecospatial perspective on the endangered status of three small, unprotected units (WQ, SL, and DSS (GH)). For the SL and DSS (GH) units, the fragmented configuration of internal habitat hotspots is a direct manifestation of their primary threats: habitat loss and fragmentation. In contrast, the WQ unit, which comprises almost entirely non-suitable habitat, points to underlying issues of ecological niche mismatch, highlighting the pressing need for exsitu conservation interventions.

### 3.4. Study Limitations and Future Directions

This study has several limitations. First, the species distribution data rely primarily on historical records and field surveys. However, field surveys indicate that wild populations of *Cycas panzhihuaensis* are virtually nonexistent outside the *Cycas panzhihuaensis* National Nature Reserve and the Pudu River Nature Reserve. Future research should expand the survey scope and validate or supplement the existing data through more systematic field censuses.

Second, although the MaxEnt model effectively identifies environmentally suitable habitats, it is essentially a mechanistic correlative model based on environmental matching, predicting the potential distribution of the species rather than the realized distribution determined by dispersal capacity and establishment success. Therefore, this study did not assess whether *C. panzhihuaensis* can actually reach and colonize these predicted suitable areas. The species’ limited seed dispersal capacity is precisely a key ecological bottleneck constraining its population recruitment [57]. Future studies should integrate seed dispersal experiments, population addition intensity observations, or genetic connectivity analyses to construct a comprehensive assessment framework that combines habitat suitability, dispersal accessibility, and population establishment probability.

Third, although this study successfully validated the correspondence between Evolutionarily Significant Units and conservation hotspots, it did not explicitly incorporate genetic diversity parameters (e.g., private allele richness) as a core conservation value layer into the spatial conservation prioritization framework. Consequently, the identification of conservation priority areas was based primarily on habitat status rather than genetic value, potentially overlooking regions with moderate habitat conditions but critical genetic contributions. The observed spatial alignment between the habitat hotspots identified in this study and independent genetic findings is encouraging; however, future research should endeavor to directly integrate habitat assessment workflows with population genetic models. Such integration holds promise for simulating and mapping “habitat threat–genetic response” risk surfaces, thereby advancing conservation decision-making from “where to protect” to “why to protect and how to prevent genetic erosion”.

## 4. Materials and Methods

### 4.1. Study Area

**Figure 6:**
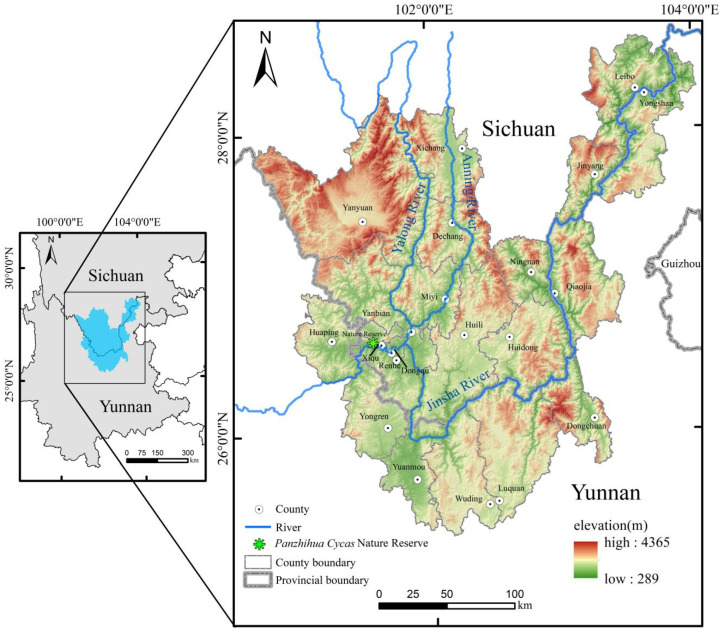
Location and topography of the study area and the *C. panzhihuaensis* Nature Reserve.

The natural distribution range of *C. panzhihuaensis* is primarily located in the arid valley regions of the Jinsha River Great Bend—spanning the border of Sichuan and Yunnan provinces—as well as the valleys of its first-order tributary, the Yalong River, and its second-order tributary, the Anning River [7]. The study area was selected based on the historical distribution records of *C. panzhihuaensis* combined with its known natural range. The finalized scope encompasses a total of 21 county-level administrative regions, including Yanyuan, Xichang, Dechang, Huili, Huidong, Ningnan, Leibo, and Jinyang counties in Liangshan Yi Autonomous Prefecture, Sichuan Province; the entire territory of Panzhihua City; as well as Huaping, Yuanmou, Yongren, Wuding, Luquan, Dongchuan District, Qiaojia, and Yongshan counties in Yunnan Province. Geographically, the area spans approximately 25°20′ N–28°36′ N and 100°42′09″ E–104°01′ E (Figure 6). The study area covers an area of 52,013.32 km^2^. Located in the lower reaches of the Jinsha River Basin, the region is characterized predominantly by mountainous and hilly terrain, with rugged landscapes, deeply incised valleys, and low-lying, enclosed topography. The foehn effect is pronounced, resulting in a dry and hot climate. The area experiences no winter throughout the year, with summers lasting over six months. The mean annual temperature ranges from 20 to 22 °C, the accumulated temperature ≥ 10 °C is 7500 °C, the mean temperature of the hottest month ranges from 25 to 28 °C, the extreme maximum temperature reaches 41 °C, the mean temperature of the coldest month is 13 °C, and the extreme minimum temperature is 0 °C. Annual precipitation is 800–1000 mm, concentrated between June and September, accounting for 90% of the total. Annual evaporation exceeds three times the annual precipitation, with the driest period occurring from March to May, during which relative humidity falls below 40%. The area exhibits typical dry-hot valley geomorphology [58]. The region supports valley-specific plant communities, primarily comprising grasslands and succulent thorny shrubs, which form distinctive types of semi-savanna and savanna vegetation [59]. The coverage of tall trees is low, and the ecosystem is fragile.

### 4.2. Data Sources and Processing

#### 4.2.1. Occurrence and Distribution Data of *C. panzhihuaensis*

The distribution data for *C. panzhihuaensis* used in this study were compiled from literature review, herbarium specimen queries, and field surveys. We referenced dried specimens from multiple platforms, including the Chinese Virtual Herbarium [60], the National Specimen Information Infrastructure [61], and the Sichuan Province Nature Reserve Specimen Platform [62]. A total of 72 distribution points were initially collected. Data from GBIF [63] show a high degree of overlap with the occurrence points we collected, exhibit significant spatial uncertainty, and lack sufficient specimen images or habitat descriptions for verification. This makes it challenging for us to effectively distinguish and exclude cultivated individuals or introduction points from non-native habitats. Therefore, GBIF data were not utilized in this study. To minimize bias caused by spatial clustering, the occurrence data were processed using the “Spatially Rarefy Occurrence Data for SDMs” tool in SDMToolbox. When two distribution points were located within a distance of less than 1 km, only one point was retained [25,27]. Fifty-eight points were ultimately retained for use in this study.

#### 4.2.2. Environmental Data

Drawing on the research of relevant experts and scholars [18,64] and building upon our team’s prior studies [28], we focus specifically on recent environmental characteristics that reflect current habitat conditions and anthropogenic disturbances. Regarding climatic variables, we employed key monthly and annual average data from 2020 rather than long-term climatic averages (such as WorldClim bioclimatic variables). This decision was primarily made to maintain temporal consistency with the anthropogenic threat sources in the InVEST model, ensuring coherence in the coupled multi-model analysis across the time dimension; it also helps in identifying the vulnerability of species under recent climate change and reduces overlap with recent work. Our team’s prior research [28] has already conducted significant work based on long-term bioclimatic variables. Building on the species occurrence data from that study, this research refines the temporal resolution to 2020, aiming to examine and reveal the impact of temporal scale selection on species distribution predictions and habitat assessment outcomes. Additionally, this study further enhances the spatial resolution of the data (standardized to 30 m) and expands soil indicators (total nitrogen, total phosphorus, total potassium) and representations of anthropogenic disturbances (population density and land use types), thereby achieving a dual improvement in the variable system in terms of spatiotemporal precision and ecological representation.

Climatic variables included the mean temperature and precipitation for the year 2020, as well as the precipitation, temperature, and mean land surface temperature for January and July 2020. These climatic data, along with soil data for total nitrogen, total phosphorus, and total potassium, were sourced from the National Tibetan Plateau Data Center [65] at a resolution of 30″ (approximately 1 km). Topographic variables comprised elevation, slope, and aspect. The elevation data, obtained at a 30 m resolution, were downloaded from the Geospatial Data Cloud of the Chinese Academy of Sciences [66] and extracted via a mask. Slope and aspect data were derived from the Digital Elevation Model (DEM) using the slope and aspect tools in ArcGIS10.8. The 2020 population density data, at a 100 m resolution, were sourced from WorldPop [67]. Soil type data were obtained from the Soil Sub-center of the National Earth System Science Data Center [68] at a 30 m resolution.

Land use data were based on the 10 m resolution global land use data for 2020 from the European Space Agency [69]. This dataset provides 11 classes defined using the Land Cover Classification System (LCCS) developed by the United Nations Food and Agriculture Organization. Additionally, railways, highways, main roads, county roads, village roads [70], and mining points were incorporated as threat sources in the InVEST model for habitat quality analysis.

#### 4.2.3. Data Processing

**Figure 7:**
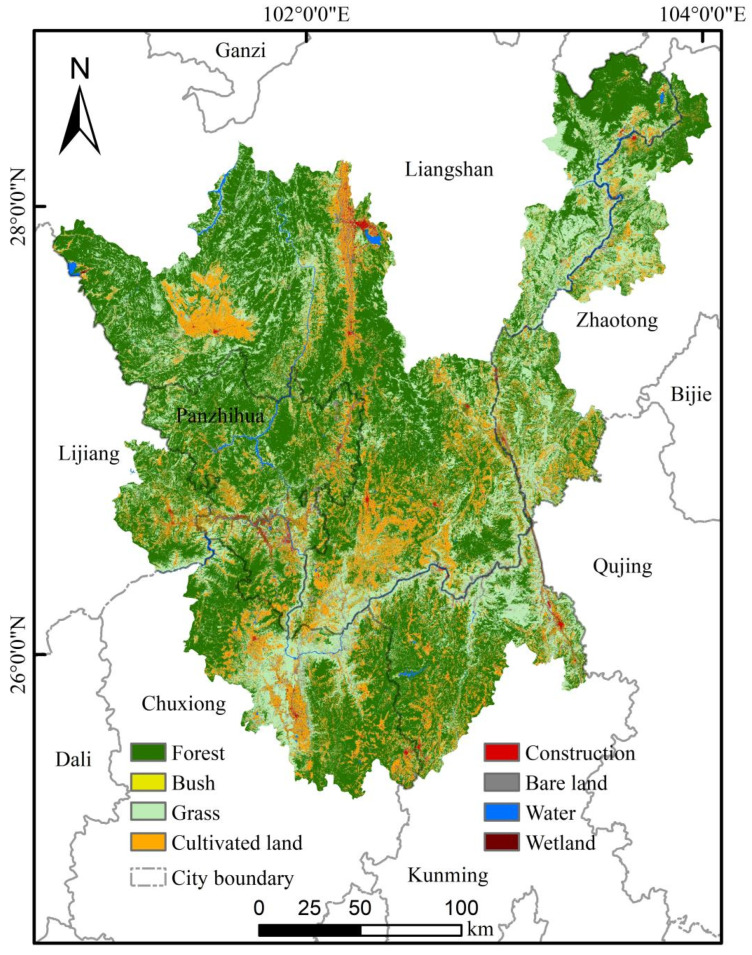
Land Use and Land Cover of the study area in 2020.

A 500 m buffer zone was created around the vector boundary of the study area to define the data processing extent. Land use types within the study area, which serve as habitat types for *C. panzhihuaensis*, were extracted using the “Extract by Mask” function. These habitat types include forest, bush, grass, cultivated land, construction, bare land, water, and wetland. Subsequently, the corresponding raster data for these land use types were collected (Figure 7).

All data were projected into the unified coordinate system WGS_1984_UTM_Zone_47N. Within the defined 500 m buffer processing extent, the resolution of various data layers was standardized. Specifically, for the climatic variables and the data on total nitrogen, total phosphorus, total potassium, and population density, resolution adjustment was performed using the Ordinary Kriging interpolation method in ArcGIS 10.8 for downscaling purposes [71]. Land use data, soil type data, and topographic factor data were resampled to unify all data at a 30 m × 30 m resolution. This resampling was a mandatory preprocessing step to enable the operation of all environmental raster layers within the MaxEnt model. The processed data were then clipped using the vector boundary of the study area as the mask to ensure that the spatial extents of all environmental factor layers were strictly consistent. Subsequently, these data were converted to ASCII format for compatibility with the MaxEnt model. To mitigate potential multicollinearity among the environmental variables, the Pearson correlation coefficient and the Variance Inflation Factor (VIF) were calculated using the Band Collection Statistics tool. Variables exhibiting both an |r| > 0.8 and a VIF > 10 were removed from the analysis [19,72]. Fourteen environmental variables were ultimately selected following the screening process. These fourteen environmental variables—specifically, mean land surface temperature in January, precipitation in January, mean land surface temperature in July, precipitation in July, mean annual precipitation, population density, total nitrogen, total phosphorus, total potassium, soil type, LULC, elevation, slope, and aspect—were used to construct the potential suitable habitat distribution model for *C. panzhihuaensis* in MaxEnt.

### 4.3. MaxEnt-Based Prediction of Suitable Habitat

This study employed the MaxEnt model to predict the suitable habitat areas for *C. panzhihuaensis* under the combined influence of the current environmental factors. The Jackknife method within the model was used to evaluate the contribution of each environmental variable to the prediction outcome. The species occurrence data were randomly partitioned, with 75% of the data points allocated as the training set for model calibration, and the remaining 25% reserved as the test set to validate the model’s accuracy [72]. The model was run 10 times with repetition, while all other parameters were set to their default values [73]. The model’s accuracy was evaluated using the Area Under the Curve (AUC) value derived from the Receiver Operating Characteristic (ROC) curve. The AUC value ranges between 0 and 1, with values closer to 1 indicating higher predictive accuracy. The predictive performance was categorized as follows: a failure for 0.0 < AUC ≤ 0.6; poor for 0.6 < AUC ≤ 0.7; fair for 0.7 < AUC ≤ 0.8; good for 0.8 < AUC ≤ 0.9; and excellent for 0.9 < AUC ≤ 1.0 [29].

### 4.4. The InVEST Habitat Quality Assessment

The Habitat Quality module of the InVEST model was used to determine the habitat quality within the study area. The resulting values range from 0 to 1, where higher values indicate a more stable ecosystem and superior habitat quality [74].

Habitat quality was determined by calculating the degree of habitat degradation based on the influence of threat sources on the habitat, and subsequently integrating this degradation level with habitat suitability [75]. Specifically, a sensitivity relationship was established between LULC (Land Use and Land Cover) and threat factors. The habitat quality index, ranging from [0, 1] with higher values indicating superior habitat quality, was calculated by integrating the distance between different habitats and threat factors, their respective weights, and the decay pattern of threat influence.

Habitat degradation refers to the degree of alteration imposed on a habitat by human-induced disturbances, representing the extent of degradation caused by threat factors. A higher value of habitat degradation indicates a greater level of threat posed to the habitat by these factors [76,77]. The model assesses the extent of external environmental impact on different regions based on their sensitivity to threats and their proximity to threat sources, thereby determining the level of habitat degradation risk. There are two types of influence that threat source rasters exert on LULC rasters: linear decay and exponential decay.

Based on the specific conditions of the study area and field survey data, the following threat sources were selected: construction, farmland, water, bare land, mining sites, railways, expressways, city roads, county roads, and village roads. The parameterization of these threats (e.g., their maximum influence distances and weights) was conducted with reference to relevant scholarly literature [22,76,77] and the model user manual [78]. Based on the above, the relevant parameters for the threat sources (Table 5), the habitat suitability for different LULC, and the sensitivity of each habitat type to the threat factors (Table 6) were determined.

### 4.5. Aggregative Computing for Conservation Hotspots

The MaxEnt model results were classified into four suitability categories: non-suitable, low-suitable, medium-suitable, and high-suitable areas [18]. The habitat quality results from the InVEST model were categorized into three levels: low, medium, and high [79]. An overlay analysis was performed between the two result layers [80]; nine overlap areas were identified between the suitable habitat zones and the habitat quality regions [26].

Habitat conservation hotspots, coldspots, and transitional zones were defined based on the intersection of habitat suitability and habitat quality (hereafter ‘quality’) classes: Hotspots were designated as areas with either: High suitability-low/medium/high quality, and medium suitability-high quality. Coldspots were defined as areas with low suitability-low quality, and low suitability-medium quality. Transitional zones comprised all other combinations, specifically areas with low suitability-high quality, medium suitability-low quality, and medium suitability-medium quality [73].

To determine the spatial aggregation of the identified habitat conservation hotspots, we employed the global Moran’s *I* index of spatial autocorrelation [81]. The value of Moran’s *I* ranges from −1 to 1. A value significantly greater than 0 indicates a clustered spatial pattern, with values closer to 1 denoting stronger clustering. Conversely, a value significantly less than 0 suggests a dispersed pattern, while a value around 0 implies a random spatial distribution. When calculating, the spatial threshold was set to 1000 m [82]. Considering the highly fragmented habitat in the dry-hot valleys, a 1000 m threshold was adopted primarily based on the limited seed dispersal ability of *C. panzhihuaensis* in order to define a locally relevant and ecologically significant spatial dependence range.

## Figures and Tables

**Table 1:** Contribution and permutation importance of 14 environmental variables (2020) in MaxEnt.

Variables	Description and Unit	Percent Contribution (%)	Permutation Importance (%)
MLST_Jan	Mean land surface temperature in January (°C)	36.1	41.3
Prec_Jan	Precipitation in January (0.1 mm)	12	10.1
Prec_Jul	Precipitation in July (0.1 mm)	10	16.1
MLST_Jul	Mean land surface temperature in July (°C)	9.6	11.9
Population density	Number of individuals per grid cell (people/900 m^2^)	7.8	1.2
TK	Total potassium	6	3.6
TN	Total nitrogen	5.3	7.1
Soil type	Soil type	4.4	0.3
MAP	Mean annual precipitation (0.1 mm)	3.2	3.2
TP	Total phosphorus	2.2	1.6
Elev	Elevation (m)	1.6	2.1
Aspect	Aspect	0.9	0.9
Slope	Slope (°)	0.5	0.2
LULC	Land Use and Land Cover	0.4	0.3

**Table 2:** Area and Proportion of Different Habitat Quality Levels.

Habitat Quality Classes	Value Range	Land Area (km^2^)	Percentage (%)
Low quality	0–0.24	11,633.87	20.75
Medium quality	0.24–0.69	44,289.28	79.00
High quality	0.69–1.00	141.45	0.25

**Table 3:** Categories and Proportions of Habitat Degradation.

Habitat Degradation Classes	Value Range	Land Area (km^2^)	Percentage (%)
Low Degradation	0–0.12	14,428.96	25.73
Medium Degradation	0.12–0.22	27,006.33	48.17
High Degradation	0.22–0.66	14,629.31	26.10

**Table 4:** Distribution and Proportion of Hotspots.

County	County Area (km^2^)	Hotspots (km^2^)	Area Occupied by the County (%)	Area Occupied by Hotspots (%)
Dechang	2302.25	35.93	1.56	4.49
Dongchuan	1875.90	6.59	0.35	0.82
Dongqu	165.96	35.83	21.59	4.48
Huaping	2142.43	49.16	2.29	6.14
Huidong	3225	13.79	0.42	1.72
Huili	4541.26	43.12	0.94	5.39
Jinyang	1587.32	8.58	0.54	1.07
Leibo	2840.71	3.93	0.13	0.49
Luquan	4244.82	2.15	0.05	0.26
Miyi	2114.37	40.95	1.93	5.12
Ningnan	1676.51	126.22	7.52	15.78
Renhe	1734.11	132.06	7.61	16.51
Wuding	2952.18	1.29	0.04	0.16
Xichang	2663	2.35	0.08	0.29
Xiqu	121.67	51.66	42.45	6.46
Yanbian	3282.57	128.93	3.92	16.12
Yanyuan	8432.03	82.55	0.97	10.32
Yongren	2152.77	0.01	0	0
Yongshan	2788.92	9.88	0.35	1.23
Yuanmou	2026.08	5.23	0.25	0.65
Qiaojia	3208.46	19.43	0.6	2.42

**Table 5:** Threat factor weight and influence distance.

Threat	Max Distance (km)	Weight	Decay
Construction	10	1	exponential
Farmland	5	0.6	linear
Water	0.1	1	linear
Bare land	2	0.3	linear
Mining	10	0.5	linear
Railway	8	0.8	exponential
Expressway	8	0.8	exponential
City road	6	0.6	exponential
County road	4	0.5	exponential
Village road	2	0.3	exponential

**Table 6:** Habitat suitability of different LULC and sensitivity to threat factors.

	LULC	Forest	Bush	Grass	Farmland	Construction	Bare Land	Water	Water Land
	Habitat suitability	0.5	1	0.6	0	0	0	0	0
**Threat factor**	Construction	0.9	0.8	0.7	0.2	0	0.1	0.4	0.6
	Farmland	0.8	0.7	0.6	0	0.1	0.2	0.5	0.7
	Water	0.9	0.9	0.9	0.2	0.3	0.2	0	0.1
	Bare land	0.7	0.9	0.9	0.4	0.1	0	0.2	0.5
	Mining	0.8	0.9	0.7	0.3	0	0.2	0.9	0.9
	Railway	0.7	0.8	0.6	0.4	0	0.3	0.3	0.6
	Expressway	0.7	0.8	0.6	0.4	0	0.3	0.3	0.3
	City road	0.7	0.8	0.6	0.4	0	0.3	0.3	0.6
	County road	0.5	0.6	0.5	0.3	0	0.2	0.2	0.4
	Village road	0.3	0.4	0.3	0.2	0	0.1	0.1	0.2

## Data Availability

The data generated during this study are available from the first author upon reasonable request. The data are not publicly available due to ongoing research.

## Supplementary Materials

The following supporting information can be downloaded at: https://www.mdpi.com/article/10.3390/plants15040670/s1, Figure S1: The habitat of *C. panzhihuaensis* and wild plants. (a) Large-scale habitat. (b) Small-scale habitat. (c,d) *C. panzhihuaensis* plants. Figure S2: AUC curve. Figure S3: Overlap between ESU units and this study.
