# Supplementary material for: Integrating Habitat Suitability and Quality Assessments to Identify Conservation Priorities for Cycas panzhihuaensis
Source: Plants (Basel). 2026 Feb 23;15(4):670. doi: 10.3390/plants15040670 (PMC12943847; doi:10.3390/plants15040670)
Supplement: Supplementary file 1 [file plants-15-00670-s001.zip › plants-4111516-Supplementary-Materials.pdf]

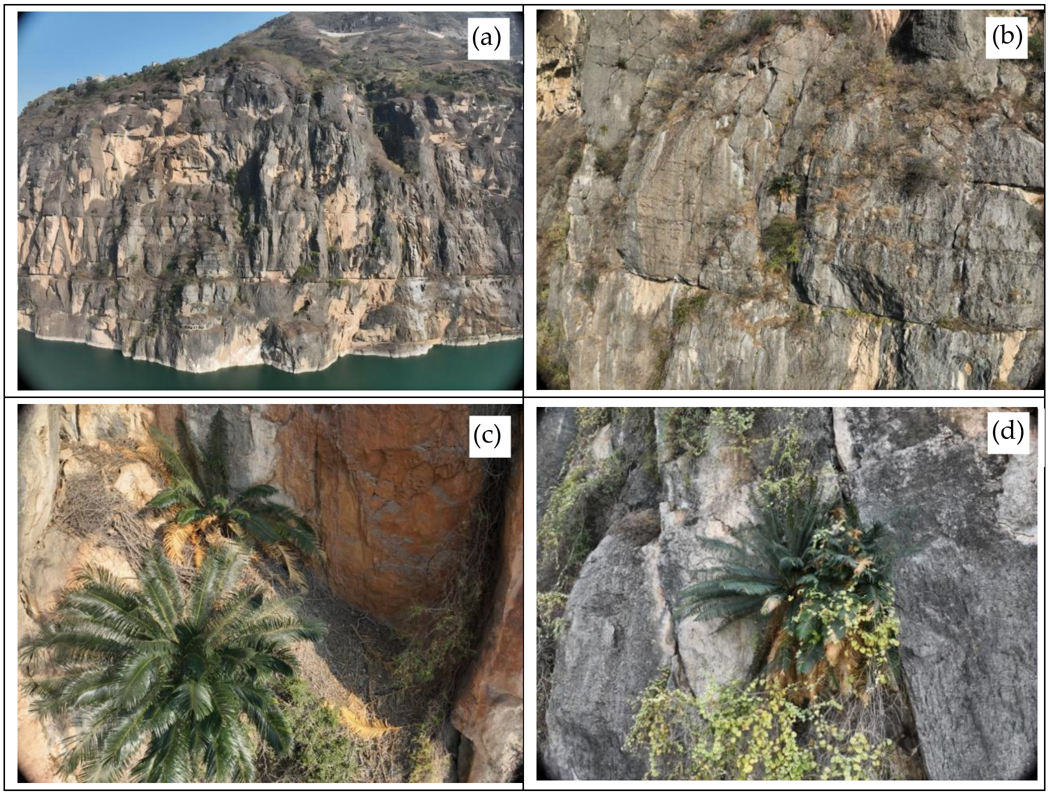

Figure S1. The habitat of *C. panzhihuaensis* and wild plants. **(a)** Large-scale habitat**. (b)** Small-scale habitat. **(c)-(d)** *C. panzhihuaensis* plants.

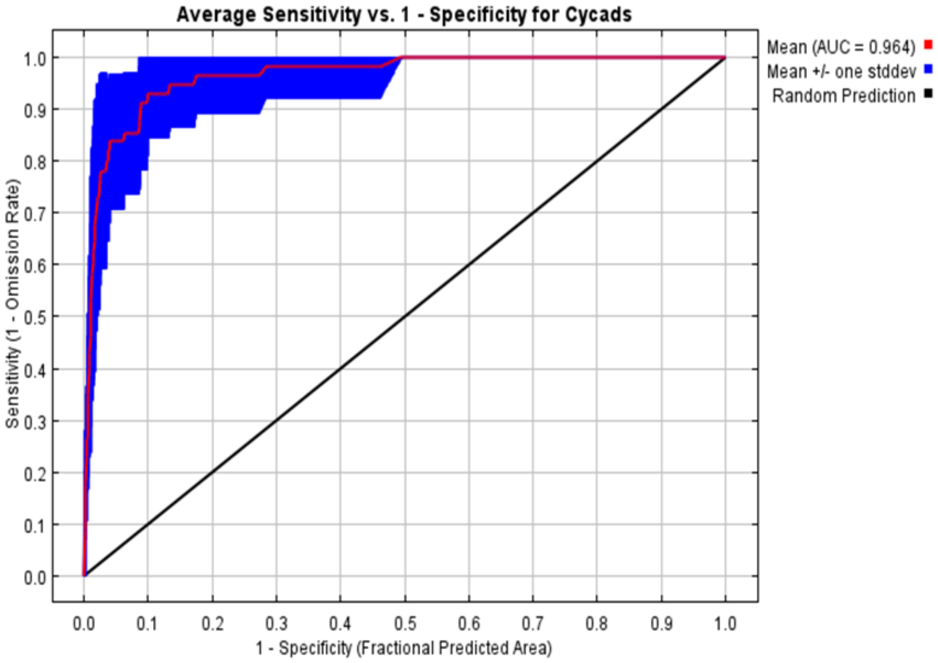

Figure S2. AUC curve.

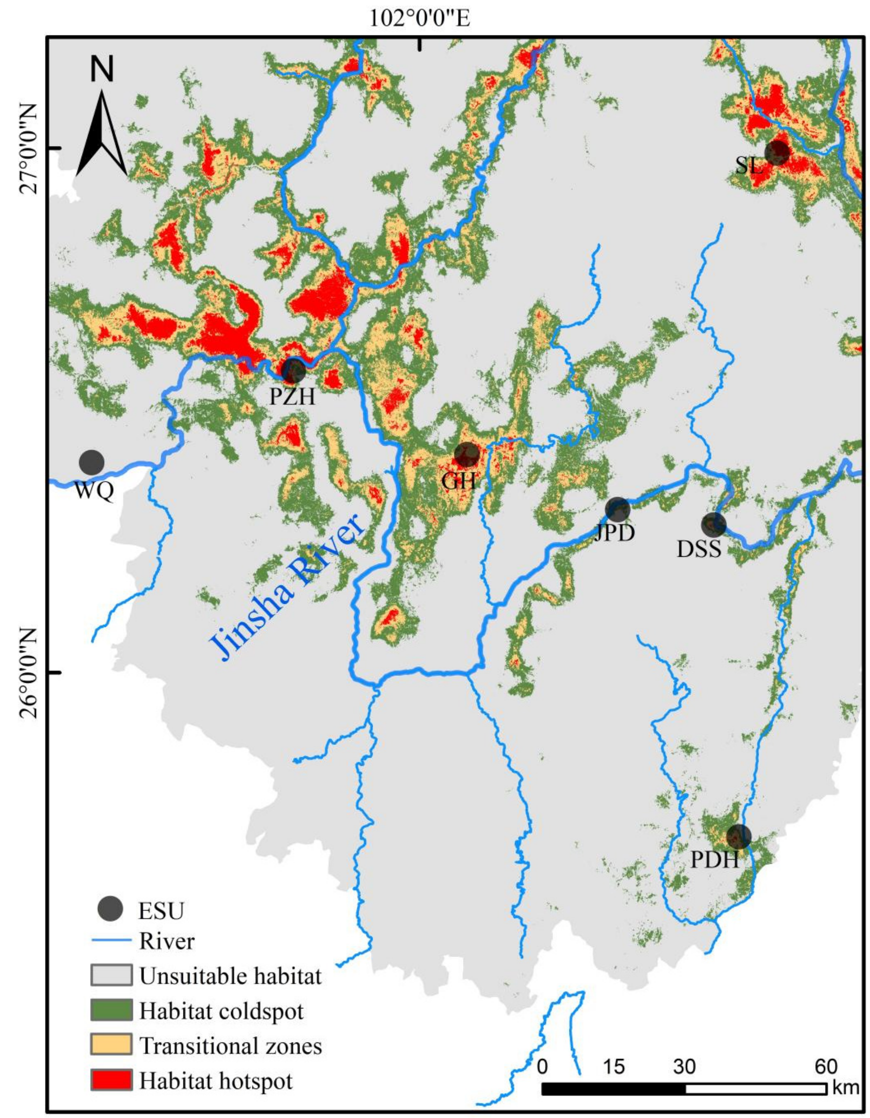

Figure S3. Overlap between ESU units and this study.
